# Blue light modulates the interactive effects of far-red light and day–night temperature difference on the growth, morphology and physiology of arugula and lettuce

**DOI:** 10.3389/fpls.2025.1721040

**Published:** 2025-12-03

**Authors:** Awais Ali, Sang Jun Jeong, Shuyang Zhen, Giacomo Cocetta, Joseph Masabni, Genhua Niu

**Affiliations:** 1 Texas A&M AgriLife Research and Extension Center, Texas A&M University, Dallas, TX, United States; 2 Department of Agricultural and Environmental Sciences, Universita Degli Studi Di Milano, Milan, Italy; 3 Division of Horticultural Science, Gyeongsang National University, Jinju, Republic of Korea; 4 Department of Horticultural Sciences, Texas A&M University, College, Station, TX, United States

**Keywords:** indoor farming, morphological modification, photon capture, cryptochromes, phytochromes, secondary metabolites, shade avoidance syndrome

## Abstract

**Introduction:**

Far-red (FR; 700–800 nm) light and the difference between day and night temperature (DIF) interactively regulate plant morphology and growth. FR light and +DIF synergistically promote stem elongation, often reducing leaf expansion and overall growth. In contrast, blue light effectively suppresses stem elongation. We hypothesized that when stem elongation is suppressed by blue light, the combination of FR light and +DIF may enhance leaf expansion, instead of stem elongation, thereby enhancing photon capture and final biomass.

**Methods:**

To determine the interactive effects among FR light, DIF, and blue light, arugula ‘Astro’ and romaine lettuce ‘Green Forest’ were grown under two blue light photon flux densities [50 (low B) and 120 (high B) μmol m^−2^ s^−1^] x three FR fractions (0.01, 0.17, and 0.33) x three DIF treatments [+8 DIF (28/20 °C), 0 DIF (24/24°C), -8 DIF (20/28°C)]. Total photon flux density was 200 μmol m^−2^ s^−1^ for the low and 270 μmol m^−2^ s^−1^ for the high blue treatments.

**Results:**

Our results showed that FR light and +DIF interactively regulated leaf expansion and stem elongation, but this effect was diminished at high blue light. In both species, under low blue light, FR light and +DIF synergistically promoted stem elongation. However, high blue light effectively suppressed the excessive stem elongation induced by FR light and +DIF, preserving their positive effects on total leaf area and biomass. In arugula, FR light and +DIF synergistically enhanced leaf expansion, rather than stem elongation, under high blue light. Morphological acclimation, such as thinner leaves under FR light and +DIF, led to a decrease in single-leaf daily carbon gain, whereas high blue light improved daily carbon gain by enhancing leaf thickness and pigment levels. Furthermore, while FR light generally reduced phenolic content and antioxidant capacity, +DIF and blue light increased flavonoid and phenolic levels as well as antioxidant capacity.

**Discussion:**

Overall, these results demonstrate that blue light is a critical determinant of whether the individual and interactive effects of FR light and +DIF are beneficial or detrimental to crop growth.

## 1 Introduction

Light not only supplies the energy required for photosynthesis but also regulates diverse aspects of plant growth and developmental processes (Demotes-Mainard et al., 2016; Wang et al., 2020). In dense vegetation, upper green leaves absorb most of the red (R; 600–700 nm) light while transmitting and reflecting a greater proportion of far-red (FR; 700–800 nm) light. This spectral filtering by the canopy decreases the R:FR ratio, which serves as a shade signal. The higher proportion of FR light triggers shade responses, including leaf expansion and elongation, stem elongation, and hyponasty, which is mediated by phytochrome (PHY) photoreceptors (Halliday and Whitelam, 2003; De Wit et al., 2016; Ballaré and Pierik, 2017). The inactivation of PHYs under FR light increases the abundance of Phytochrome-Interacting Factors (PIFs), which in turn regulate a suite of genes associated with cell expansion and hormonal signaling (Sellaro et al., 2017). In controlled environment agriculture, FR light-induced morphological changes such as increased leaf expansion have been utilized to enhance photon capture and crop yield, given the strong correlation between photon capture and biomass accumulation (Klassen et al., 2004; Park and Runkle, 2017; Meng and Runkle, 2019; Zhen and Bugbee, 2020a; Legendre and van Iersel, 2021).

In addition to light, plant morphology is influenced by temperature conditions (thermomorphogenesis) (Casal and Balasubramanian, 2019). Vegetative growth is affected by two main types of temperature cues: average temperature and diurnal temperature fluctuation, commonly referred to as the difference between day temperature and night temperature (DIF) in horticultural field. Beyond the acceleration of metabolic processes, warm temperature stimulates specific morphological changes, including leaf and stem elongation, and leaf expansion (Casal and Balasubramanian, 2019). Similarly, plants grown under positive DIF (+DIF; warmer day than night) generally showed increases in plant height, internode length, and leaf angle, whereas those under negative DIF (−DIF; cooler day than night) exhibited more compact morphology (Erwin et al., 1989; Erwin and Heins, 1995; Ohtaka et al., 2020).

Consistent with their morphological similarity to FR light response, both average temperature and diurnal temperature fluctuation (i.e., DIF) can be perceived by PHYB photoreceptors (Thingnaes et al., 2008; Jung et al., 2016; Legris et al., 2016; Ohtaka et al., 2020). Through this shared signaling mechanism, FR light interacts with the temperature cues to regulate plant morphology. For example, the combination of FR light and warm temperature synergistically promotes stem or hypocotyl elongation (Romero-Montepaone et al., 2021; Burko et al., 2022). This response, however, often occurs at the expense of leaf development, ultimately reducing total leaf area and biomass accumulation (Jeong et al., 2024a). These findings indicate that the excessive stem elongation induced by FR light and warm temperature can limit leaf growth and lead to potential yield loss. Similarly, +DIF can also enhance plant sensitivity to FR light, resulting in more pronounced stem elongation compared with −DIF (Patil and Moe, 2009; Jeong et al., 2024b). The synergistic stem elongation by FR light and +DIF are comparable to the response observed under FR light and warm temperature, potentially reducing leaf area and overall biomass. However, most studies on the interaction between FR light and +DIF have primarily focused on stem elongation, while the implications for leaf development and its subsequent effects on plant growth remaining poorly understood.

Morphological responses to FR light further depend on light intensity (Park and Runkle, 2019; Kusuma and Bugbee, 2023). For instance, higher light intensity (200 and 500 µmol m^-2^ s^-1^) effectively suppressed FR light-induced stem elongation, compared with a lower light intensity (100 µmol m^-2^ s^-1^) in lettuce (Kusuma and Bugbee, 2023). Under high light, FR light preferentially promoted leaf expansion rather than stem elongation. In these previous studies, blue light (B; 400–500 nm) intensity increased with increasing total light intensity. Blue light plays a central role in suppressing shade responses, such as leaf and stem elongation, in various crops such as lettuce, tomato, basil, and kale (Kaiser et al., 2019; Meng et al., 2019; Park and Runkle, 2019; Shin et al., 2025). Blue light suppresses shade responses by activating cryptochrome (CRY) photoreceptors, thereby inhibiting PIF activity and associated hormonal regulations (Ma et al., 2016; Procopio et al., 2016; Wang and Lin, 2020). Consequently, blue light promotes the development of a “sun-type” morphology characterized by compact growth, thick leaves, and high photosynthetic capacity (Savvides et al., 2012), whereas the absence of blue light can lead to reduced canopy light interception with epinasty (leaf rolling) and ultimately decreased biomass accumulation (Jeong et al., 2025).

Light spectra and temperature also modulate the accumulation of various photosynthetic pigments and secondary metabolites and antioxidant capacity, which are closely associated with nutritional quality and visual appearance (Shamloo et al., 2017; Zhang et al., 2021). FR light generally reduced the accumulation of chlorophylls, carotenoids, and phenolics (Li and Kubota, 2009; Stutte et al., 2009; Toledo-Ortiz et al., 2010). In contrast, exposure to blue light has been widely reported to enhance photosynthetic pigments, such as chlorophyll and carotenoids, as well as diverse secondary metabolites (Li and Kubota, 2009; Johkan et al., 2010; Salam et al., 2023). The influence of DIF on pigments and secondary metabolite accumulation is less characterized. Some studies have reported that, compared to -DIF, +DIF resulted in higher chlorophyll concentration in some species, including basil, lemon balm, and tomato (Vågen et al., 2003; Yuan, 2016).

Regulation of light spectra has become one of the key strategies for enhancing the crop productivity and nutritional quality of leafy greens. However, day-night temperature differences naturally occur with sunset and sunrise or with the turning on and off of artificial lighting, and these fluctuations vary with daily and seasonal environmental conditions. In controlled environments (e.g., indoor farms and greenhouses), DIF is also often intentionally manipulated to achieve desirable morphological traits and to improve crop yield and photosynthetic performance in various crops (Erwin et al., 1989; Erwin and Heins, 1995; Kanno and Makino, 2010; Matsuda et al., 2014). Given that light spectral conditions and temperature regimes (i.e., DIF) often coincide, it is essential to understand how these factors interact to regulate plant morphology, physiology, and overall growth. Despite their importance, the interactive effects of light spectra and DIF on plant growth, morphology, and physiology remain poorly understood. In this study, we hypothesized that the synergistic stem elongation induced by FR light and +DIF may limit leaf growth, thereby reducing crop yield. However, we further hypothesized that the inhibitory effects of blue light on stem elongation could mitigate the potential problem. The objectives of this study were 1) to investigate how FR light and DIF interact under different blue light intensities, with a focus on morphology, photosynthetic performance, pigmentation, and secondary metabolite accumulations and 2) to identify potentially optimal combinations of these environmental factors for crop yield and quality.

## 2 Materials and methods

### 2.1 Plant materials

In a glass-covered greenhouse, 450-mL square pots were filled with all-purpose soilless substrate (BM6, Berger, Saint-Modeste, QC, Canada). Romaine lettuce (*Lactuca sativa*) ‘Green Forest’ (1 seed/pot) and arugula (*Eruca sativa*) ‘Astro’ seeds (3 seeds/pot) were used in this experiments (Johnny’s Selected Seeds, Winslow, ME, USA). Six days after sowing, both romaine lettuce and arugula seedlings were transferred to growth chambers (Environmental Growth Chambers, Chagrin Falls, OH, USA). For arugula, seedlings were chosen based on their homogeneity and then thinned to one plant per pot. A nutrient solution comprising 150 mg L^−1^ N, prepared using a water-soluble fertilizer (21 N-2.2 P-16.6 K; Peters 21–5–20; The Scotts Company, Marysville, OH, USA), was manually applied to the plants as needed. Lettuce and arugula were grown together in the same experimental units.

### 2.2 Temperature and light treatments

**Figure 1:**
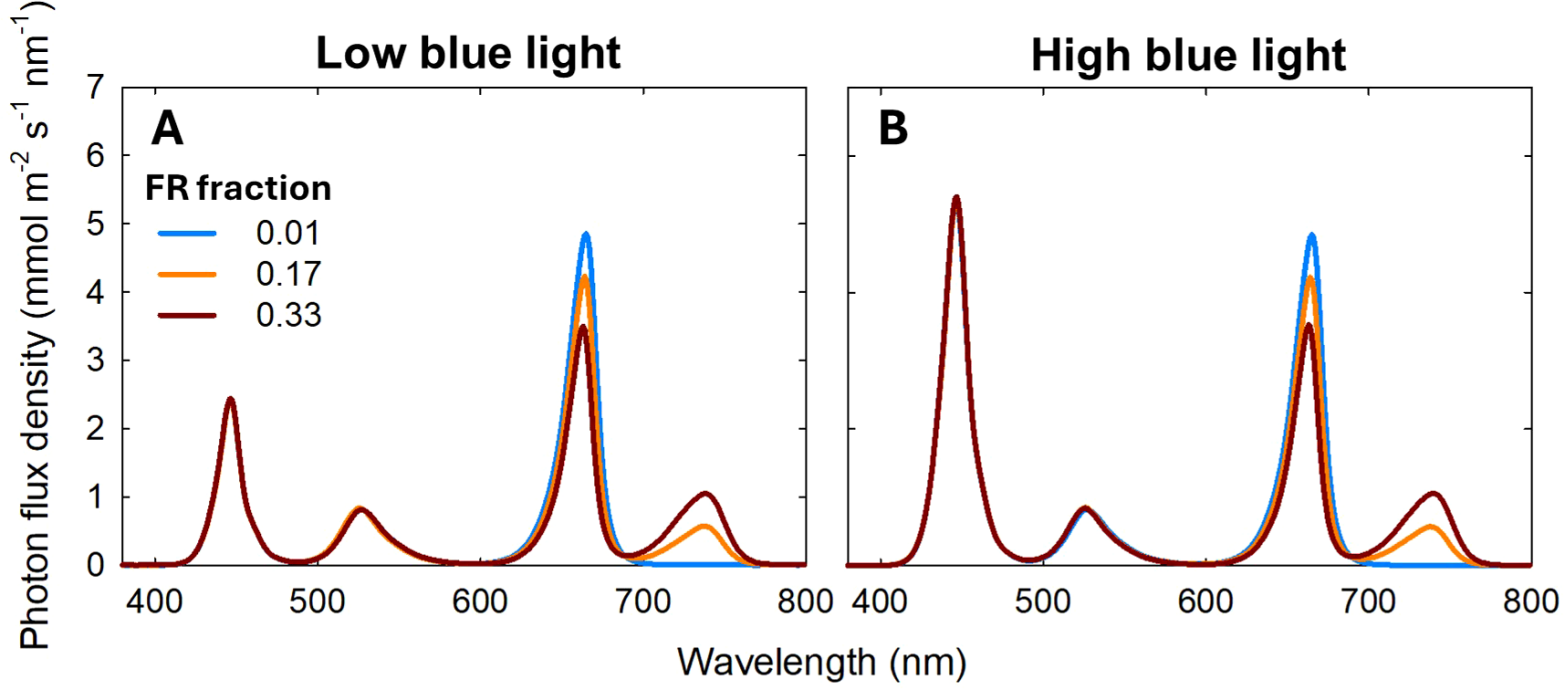
Spectral distributions of six light treatments consisted of blue (400–500 nm), green (500–600 nm), red (R; 600–700 nm), and far-red (FR; 700–800 nm) photons delivered by light-emitting diodes. Two different levels of blue light [50 (low blue light; **(A)** and 120 (high blue light; **(B)** μmol m^−2^ s^−1^], three different far-red (FR) fractions [FR/(red (R) + FR); 0, 0.17, and 0.33] were used. The total photon flux density was 200 μmol m^−2^ s^−1^ for low blue light and 270 μmol m^−2^ s^−1^ for high blue light conditions.

This study included 18 treatments formed by combining three temperature regimes with various light spectra (**Table 1**). Three growth chambers (2.9 m × 1.4 m × 2.4 m; length × width × height; Growtainers^®^; Sycamore, IL, USA) with temperature set at 20, 24, and 28°C, respectively, were used. Three DIF conditions were used: -8 DIF, 0DIF, and +8 DIF. To create -8 DIF and +8 DIF, plants were moved between the 20°C and 28°C growth chambers twice daily: once within 30 minutes before the start of the light period and again at the beginning of the dark period. The 0DIF condition was maintained at a constant temperature of 24°C throughout both the light and dark periods. All treatments had a 12/12 h light/dark photoperiod (08:00 – 20:00). Each chamber was divided into six sections (0.7 m x 0.7 m x 0.7 m; length x width x height) using reflective cardboard to accommodate spectral treatments consisting of blue, green (G; 500–600 nm), R and FR light. A total of six spectral treatments were used under each temperature condition: three with low blue photon flux density and three with high blue photon flux density. In the low blue treatments (50 μmol m^-2^ s^-1^ of blue light), the total photon flux density (TPFD; 400–800 nm) was set to 200 μmol m^−2^ s^−1^. Three spectral treatments were applied: B_50_G_30_R_120_FR_0,_ B_50_G_30_R_100_FR_20_ and B_50_G_30_R_80_FR_40_, which corresponded to FR fractions [FR/(R +FR)] of 0.01, 0.17, and 0.33, respectively (**Figure 1**). The subscript number represents the photon flux density in μmol m^−2^ s^−1^. In the high blue light condition (120 μmol m^-2^ s^-1^ of blue light), TPFD was set to 270 μmol m^−2^ s^−1^. The three spectral treatments were B_120_G_30_R_120_FR_0_, B_120_G_30_R_100_FR_20_ and B_120_G_30_R_80_FR_40_, corresponding to FR fractions of 0.01, 0.17, 0.33, respectively (**Figure 1**). Two TPFD levels- 200 μmol m^−2^ s^−1^ in treatments with low blue light and 270 μmol m^−2^ s^−1^ in treatments with high blue light, were implemented to maintain a consistent phytochrome photoequilibrium (PPE) across spectral treatments. The spectral treatments were established using PHYTOFY^®^ RL LED research lighting system (Osram, Munich, Germany). Photon flux density at plant height (30 cm below the LEDs) was measured at fourteen locations within each treatment area using a spectroradiometer (PS100; Apogee Instruments, Logan, UT, USA). A type-E thermocouple was installed in each chamber section and connected to a data logger (CR1000; Campbell Scientific, Logan, UT, USA) to measure air temperature every 5s and the average of every 20 min was recorded. The average air temperature of chambers was 21.1, 24.5, and 28.5°C for replicate 1, 20.6, 23.9, and 28.0°C for replicate 2, and 20.2, 24.1, and 28.8°C for replicate 3 (**Supplementary Figure 1**). To minimize spatial variations in light intensity, plants within each treatment were rotated randomly every day.

**Table 1:** Temperature, light spectral characteristics, and photon flux density used.

TPFD^z^ (µmol m^-2^ s^-1^)	Temperature [Day/Night (°C)]	B (µmol m^-2^ s^-1^)	G (µmol m^-2^ s^-1^)	R (µmol m^-2^ s^-1^)	FR (µmol m^-2^ s^-1^)	B (%)	G (%)	R (%)	FR (%)	FR fraction [FR/(R+FR)]	Estimated PPE^y^
200	20/28 (-8 DIF^x^)	50	30	120	0	25	15	60	0	0.01	0.87
		50	30	100	20	25	15	50	10	0.17	0.83
		50	30	80	40	25	15	40	20	0.33	0.78
	24/24 (0 DIF)	50	30	120	0	25	15	60	0	0.01	0.87
		50	30	100	20	25	15	50	10	0.17	0.83
		50	30	80	40	25	15	40	20	0.33	0.78
	28/20 (+8 DIF)	50	30	120	0	25	15	60	0	0.01	0.87
		50	30	100	20	25	15	50	10	0.17	0.83
		50	30	80	40	25	15	40	20	0.33	0.78
270	20/28 (-8 DIF)	120	30	120	0	44	11	44	0	0.01	0.86
		120	30	100	20	44	11	37	7	0.17	0.82
		120	30	80	40	44	11	30	15	0.33	0.77
	24/24 (0 DIF)	120	30	120	0	44	11	44	0	0.01	0.86
		120	30	100	20	44	11	37	7	0.17	0.82
		120	30	80	40	44	11	30	15	0.33	0.77
	28/20 (+8 DIF)	120	30	120	0	44	11	44	0	0.01	0.86
		120	30	100	20	44	11	37	7	0.17	0.82
		120	30	80	40	44	11	30	15	0.33	0.77

z TPFD, Total photon flux density (µmol m^-2^ s^-1^; 400 to 800 nm).

y Estimated PPE, Phytochrome photoequilibrium calculated based on Sager et al. (1988).

x DIF, Difference between day and night temperature (i.e., Daytime temperature – nighttime temperature).

Light spectra consisted of blue (B; 400–500 nm), green (G; 500–600 nm), red (R; 600–700 nm), and far-red (FR; 700–800 nm) photons from light-emitting diodes. The subscript after each waveband indicates its photon flux density in μmol m^−2^ s^−1^.

### 2.3 Data collection

#### 2.3.1 Plant morphology and growth parameters

Morphological and growth parameters were measured at harvest [20 days after treatment (DAT) for arugula and 23 DAT for lettuce]. For both lettuce and arugula, total leaf number, leaf length, leaf width, and stem length were recorded. Total leaf area was measured with a leaf area meter (LI-3100; LI-COR Biosciences, Lincoln, NE, USA). Fresh weight (FW) and dry weight (DW; determined after drying at 80°C for 7 days in a drying oven) of leaves, stems, and roots were recorded. The proportions of stem DW (%), leaf DW (%), and root DW (%) in total DW were calculated. Specific leaf area was determined by dividing total leaf area by leaf DW.

#### 2.3.2 Photosynthetic measurement

Single-leaf photosynthetic analysis was conducted on the most recently fully expanded leaves one to three days prior to harvest.

After dark-adaptation for 30 minutes using dark period (22:00 – 23:00), the minimum fluorescence (*F_o_*) was measured using a chlorophyll fluorometer (OS5p; Opti-Science, Inc., Hudson, NH, USA). Then, the maximum fluorescence (*F_m_*) was measured by applying a saturating light pulse. The maximum quantum efficiency of PSII photochemistry was calculated as *F_v_*/*F_m_*, where *F_v_* represents the variable fluorescence (*F_v_* = *F_m_* − *F_o_*). The quantum yield of PS II (*Φ_PSII_*) was calculated using the formula [*Φ_PSII_* = (*Fm’* − *F’*)/*Fm’*], where *F’* represents the steady state fluorescence and *Fm’* represents the maximum fluorescence measured in the light-adapted state (Baker, 2008).

The net CO_2_ assimilation rate (*P_net, light_*) and dark respiration rate (*R_dark_*) were measured using a CIRAS-3 portable gas exchange analyzer (CIRAS-3; PP systems, Amesbury, MA, USA) with a PLC3 leaf cuvette (25×18 mm) and a clear top chamber. *P_net, light_* and *R_dark_* were measured during the daytime (10:00 – 16:00) and nighttime (22:00 – 02:00), respectively, one to three days prior to harvest (same day for measuring chlorophyll fluorescence). The CO_2_ concentration inside the cuvette was controlled and held constant at 390 μmol mol^−1^.

Daily carbon gain at the single-leaf level was estimated by integrating carbon exchange rate over a 24-h period, using the following equation (Van Iersel, 2003; Frantz et al., 2004):


$$\text{Estimated daily carbon gain }({\text{mol CO}}_{2}{\text{ m}}^{-2}{\text{ d}}^{-1})=(P_{net,\;light}\times \text{light period}-|R_{dark}|\times \text{dark period})$$

Where the light and dark period were 12 hours each in this study. |R_dark_| represents the absolute value of the dark respiration rate. Photosynthetic analyses were conducted under the given light treatments using incident light. A spectroradiometer (PS-100) was used to confirm that the target light conditions were achieved.

#### 2.3.3 Pigments, secondary metabolites, and antioxidant capacity measurement

The youngest matured leaves were collected at midday, one day before harvest, for the measurement of photosynthetic pigments, secondary metabolites, and antioxidant capacity. Immediately after collection, the samples were immersed in liquid N_2_, homogenized using a mortar and pestle, and stored at -80°C until subsequent analysis.

To measure photosynthetic pigment, fresh plant tissue (50 mg) was placed in a 2-ml tube containing 1.5 ml pure methanol and kept out of light at 4°C for 24 hours. After extraction, samples were vortexed vigorously under low light followed by centrifugation at 10,000 rpm for 10 minutes at 10°C. Absorbance readings were taken against a pure methanol blank at four wavelengths using the Thermo Scientific Genesys 10S UV-VIS spectrophotometer: 750 nm (*A_750_* = 0 for a clear extract), 665.2 nm (chlorophyll *a* maximum in methanol), 652.4 nm (chlorophyll *b* maximum in methanol), 470 nm (carotenoids). Pigment concentrations were determined from the formulas according to Wellburn (1994), with slight modifications as follows.


$$\text{Chl a}\;({\text{µg ml}}^{-1}\;)=16.72\;\times ({\text{A}}_{665.2})\;-9.16\times ({\text{A}}_{652.4})$$

$$\text{Chl b}({\text{µg ml}}^{-1})=\;34.09\times ({\text{A}}_{652.4})\;-\;15.28\times ({\text{A}}_{665.2})\;$$

$$\text{Car}\;({\text{µg ml}}^{-1})=[1000\times ({\text{A}}_{470})-1.63\times (\text{Chl a})-104.96\times (\text{Chl b})]/221$$

Secondary metabolites and antioxidant capacity were measured as described in Dou et al. (2019). For extraction, 100 mg of fresh samples was homogenized in 0.75 ml 1% acidified methanol at 4°C in darkness. After overnight extraction the mixture was centrifuged at 10,000 g for 10 min, and the resulting supernatant was collected for subsequent analysis. For total phenolic content, a 100 μL of extract was transferred to combine with 150 μL distilled water and 750 μL 1/10 dilution Folin-Ciocalteau reagent. After 6 mins, 600 μL of 7.5% sodium carbonate (Na_2_CO_3_) was added followed by shaking for 30s and incubation at room temperature for 2 hours. Finally, absorbance was measured at 725 nm using a microplate reader (ELx800, Bioek, Winooski, VT, USA), while making sure that there were no bubbles in the microplate. Results were expressed as milligram gallic acid equivalent (GAE) per gram FW.

For total flavonoids, a 20 µL extract was placed into microplates followed by the addition of 85 µL distilled water and 5 µL 5% sodium nitrite (NaNO_2_). Mixture was kept for 6 mins to react and then 10 µL 10% aluminum chloride hexahydrate (AlCl_3_·6H_2_O) was added. After 5 mins, 35 µL of 1M sodium hydroxide (NaOH) and 20 µL distilled water were added into the mixture. The mixture was shaken vigorously, and absorbance was immediately measured at 520 nm using the EL×800 microplate. The results were expressed as mg of (+)–catechin hydrate (CE) equivalents/g of fresh weight for total flavonoid.

Likewise, antioxidant capacity was evaluated using the 2,2’-azino-bis (3-ethylbenzothiazoline-6-sulphonic acid) (ABTS) decolorization assay described by Arnao et al. (2001). A 50 µL of extracts was mixed with 950 µL ABTS solution, incubated at room temperature for 10 min. Absorbance readings were then measured at 734 nm using the microplate reader EL×800. The results were expressed as milligrams of Trolox equivalent antioxidant capacity per gram FW of arugula and lettuce.

### 2.4 Statistical analysis

This experiment was replicated three times. For each replicate, four plants (subsamples) per species per treatment were included in each of the eighteen treatments, consisting of two different blue light intensities (50 and 120 μmol m^−2^ s^−1^), three FR fraction (0.01, 0.17, and 0.33), three DIF treatments [+8DIF (28/20°C), 0DIF (24/24°C), -8DIF (20/28°C)]. A split-plot block design was employed, with temperature assigned as the main-plot factor and spectral quality as the sub-plot factor. In each replicate, the chamber temperature settings and the allocation of spectral treatments were randomized. Prior to statistical analysis, subsample data were averaged. The normality of residuals was tested using the Shapiro-Wilk test, and the homogeneity of variances was assessed using the Brown-Forsythe test based on raw data before analysis of variance (ANOVA). A three-way ANOVA was performed to evaluate the interactive effects among blue light, DIF, and FR light, while a two-way ANOVA was used to determine the interaction between DIF and FR light within each blue light level. To investigate how the interactive effects between DIF and FR light vary under different blue light intensity, the data and statistical results were visualized separately for the two blue light levels. When significant effects were detected, treatment means were separated using Duncan’s multiple range test at *p* < 0.05. All statistical analyses were conducted using the Statistical Analysis System version 9.4 (SAS Inst., Inc., Cary, NC, USA).

## 3 Results

### 3.1 Plant morphology

**Figure 2:**
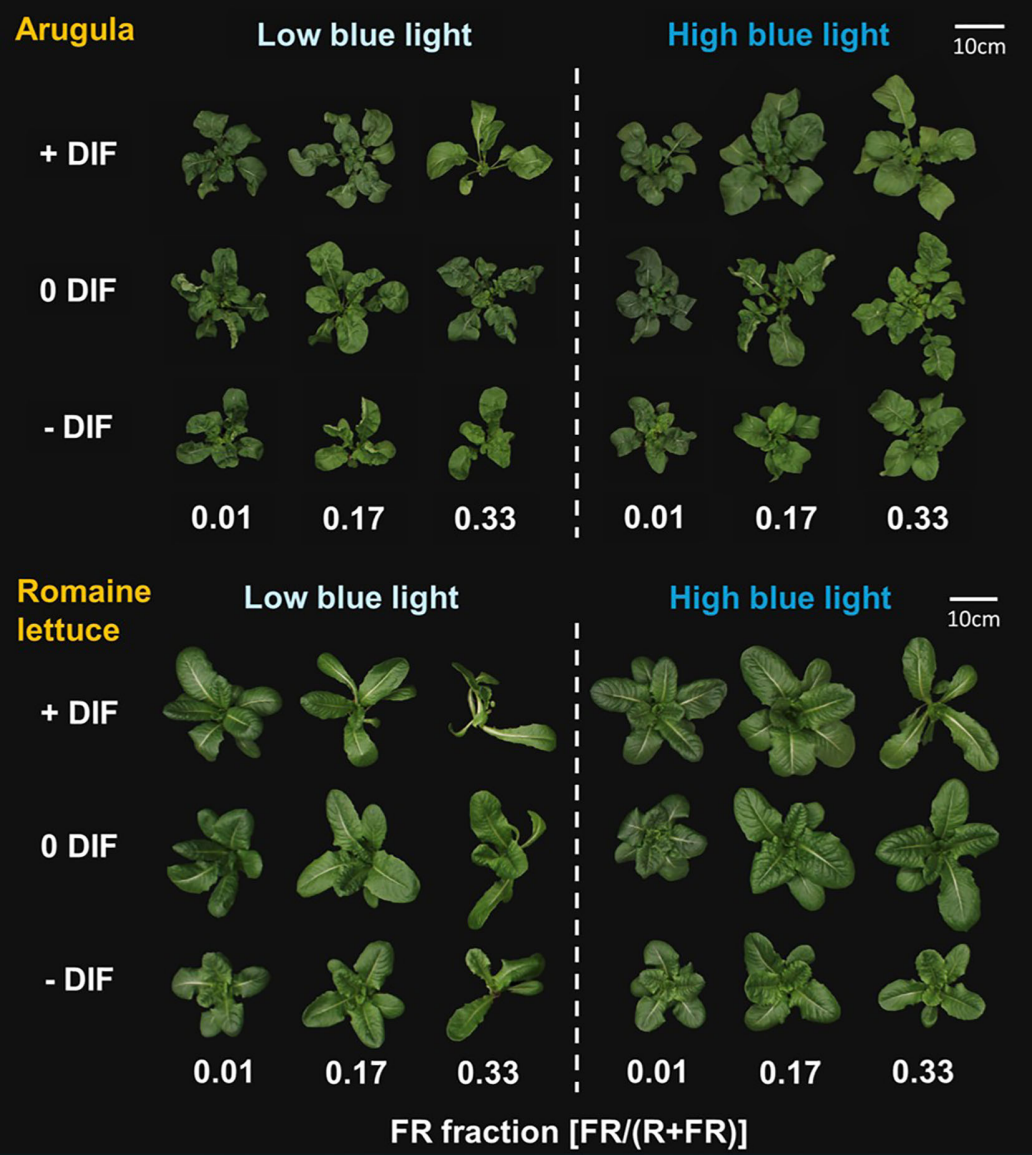
Representative plants of arugula and romaine lettuce grown under eighteen treatments composed of two different blue light intensities [50 (low blue light) and 120 (high blue light) μmol m^−2^ s^−1^] x three far-red (FR) fractions [FR/(red (R) + FR); 0, 0.17, and 0.33] x three difference (DIF) between day and night temperature treatments [+8DIF (28/20°C), 0DIF (24/24°C), -8DIF (20/28°C)].

**Figure 3:**
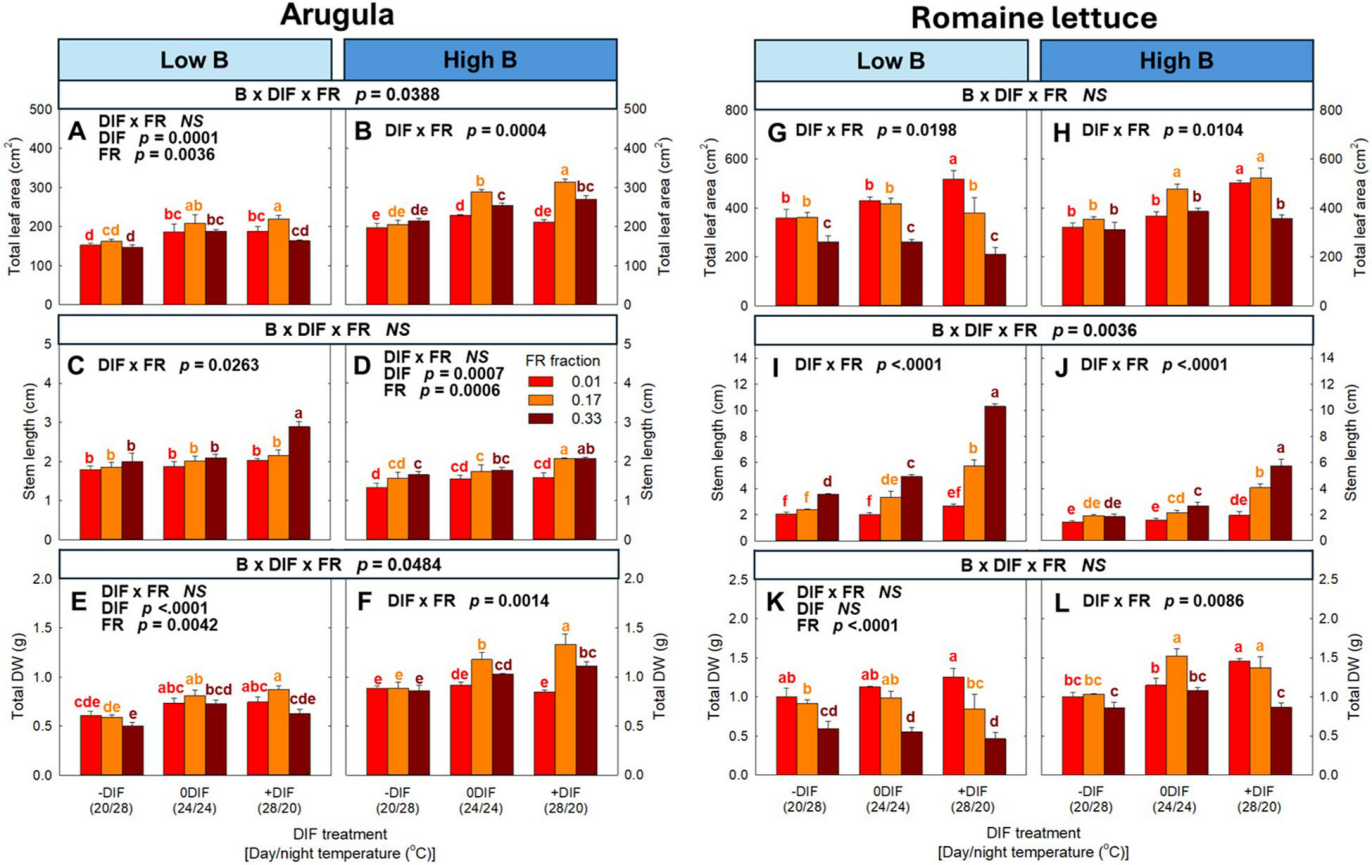
The interactive effect between light spectral quality [0.01, 0.17, and 0.33 of far-red (FR) fraction] and temperature [-DIF (difference between day and night temperature), 0DIF, +DIF] on total leaf area **(A, B)**, stem length **(C, D)** and total DW **(E, F)** of arugula and total leaf area **(G, H)**, stem length **(I, J)** and total DW **(K, L)** of romaine lettuce under low and high blue light conditions. Three-way ANOVA was conducted to assess the interactive effects among FR fraction, DIF, and blue light. To further evaluate the interactions between FR light and DIF under different blue light intensities, two-way ANOVA was performed for FR fraction and DIF within each blue light level. Different letters above the mean ± SE [n = 3; subsamples (4 plants per treatment per replicate study) were averaged before statistical analysis] indicate significant difference among the nine treatments (three FR fractions x three DIF treatments) at p < 0.05. NS stands for non-significance.

Significant interactive effects among FR light, DIF and blue light were observed in several morphological parameters in arugula and romaine lettuce (**Figures 2**, **3**; **Supplementary Figure 2**). Specifically, total leaf area in arugula and stem length in romaine lettuce showed significant three-way interactions, and stem length of arugula was also interactively regulated by these environmental factors with marginal significance (*p* = 0.0880) (**Figure 3**). Notably, the interactive effects between FR light and DIF were dependent on blue light intensity. Under low blue light, FR light tended to promote leaf expansion in arugula, and no significant interaction between FR light and DIF was observed for total leaf area. Conversely, under high blue light, FR light and +DIF synergistically increased total leaf area, showing a significant interaction. More specifically, 0.17 FR fraction led to no increase in total leaf area of arugula at -DIF, a 26% increase at 0DIF, and a 49% increase at +DIF (**Figures 3A, B**). In contrast, FR light and +DIF synergistically stimulated stem elongation under low blue light, but not at high blue light, with significant interaction between FR light and DIF only at low blue light (**Figures 3C, D**). Similarly, in romaine lettuce, the stimulative effect of FR light on stem elongation was more pronounced under low blue light than high blue light (**Figures 3I, J**). Total leaf area of romaine lettuce typically decreased with increasing FR fraction, and this reduction was more evident under +DIF with significant interaction between FR light and DIF at both low and high blue light conditions (**Figures 3G, H**).

### 3.2 Plant biomass

Similar to the morphological response observed in total leaf area and stem length, significant three-way interactions were observed in total DW and stem DW (%) in total DW in arugula, respectively (**Figure 3**; **Supplementary Figure 3**). The interaction between FR light and DIF on total DW of arugula was observed at high blue light level, but not at low blue light level. Specifically, FR light showed no significant effect on total DW of arugula at any DIF treatment under low blue light, while resulting in no significant changes at −DIF, a 28% increase at 0DIF, and a 57% increase at +DIF under high blue light (**Figures 3E, F**). In contrast, stem DW (%) in total DW showed synergistic effects between FR light and +DIF at low blue light, but not at high blue light (**Supplementary Figures 3C, D**). In romaine lettuce, total DW responded to environmental factors in a pattern similar to total leaf area (**Figures 3K, L**). The combination of FR light and +DIF synergistically decreased leaf DW (%) in total DW and increased stem DW (%) in total DW in both low and high blue light (**Supplementary Figures 3G-J**). However, the interactive effects between FR light and DIF were more pronounced under low blue light in both parameters.

### 3.3 Photosynthesis at single leaf level

**Figure 4:**
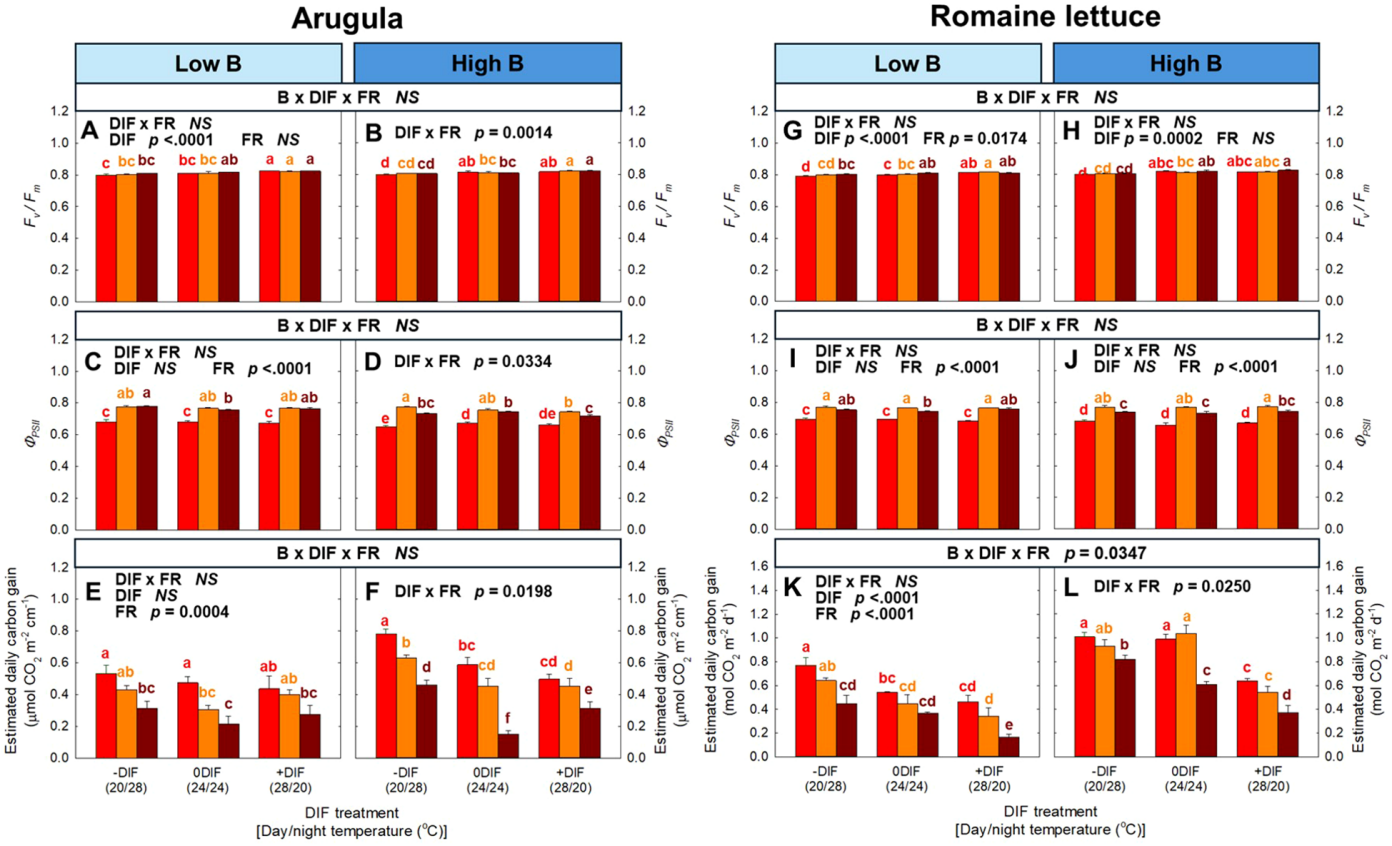
The interactive effect between light spectral quality [0.01, 0.17, and 0.33 of far-red (FR) fraction] and temperature [-DIF (difference between day and night temperature), 0DIF, +DIF] on *F_v_/F_m_***(A, B)**, *Φ_PSII_***(C, D)** and estimated daily carbon gain **(E, F)** of arugula and *F_v_/F_m_***(G, H)**, *Φ_PSII_***(I, J)** and estimated daily carbon gain **(K, L)** of romaine lettuce under low and high blue light conditions. Three-way ANOVA was conducted to assess the interactive effects among FR fraction, DIF, and blue light. To further evaluate the interactions between FR light and DIF under different blue light intensities, two-way ANOVA was performed for FR fraction and DIF within each blue light level. Different letters above the mean ± SE [n = 3; subsamples (4 plants per treatment per replicate study) were averaged before statistical analysis] indicate significant difference among the nine treatments (three FR fractions x three DIF treatments) at p < 0.05. NS stands for non-significance.

No interactive effect among FR light, DIF, and blue light was observed for the photosynthetic parameters, including *F_v_/F_m_*, *Φ_PSII_*, and estimated daily carbon gain, in arugula and romaine lettuce, except for estimated daily carbon gain in romaine lettuce (**Figure 4**). In both species, +DIF tended to increase *F_v_/F_m_*, whereas FR light generally had no significant effect (**Figures 4A, B, G, H**). *Φ_PSII_* significantly increased by FR light in both low and high blue light intensities, but the effect of DIF on *Φ_PSII_* was minimal in both arugula and romaine lettuce (**Figures 4C, D, I, J**). Estimated daily carbon gain was typically decreased with increasing FR fraction (**Figures 4E, F, K, L**).

### 3.4 Pigments, secondary metabolites, and antioxidant capacity

**Figure 5:**
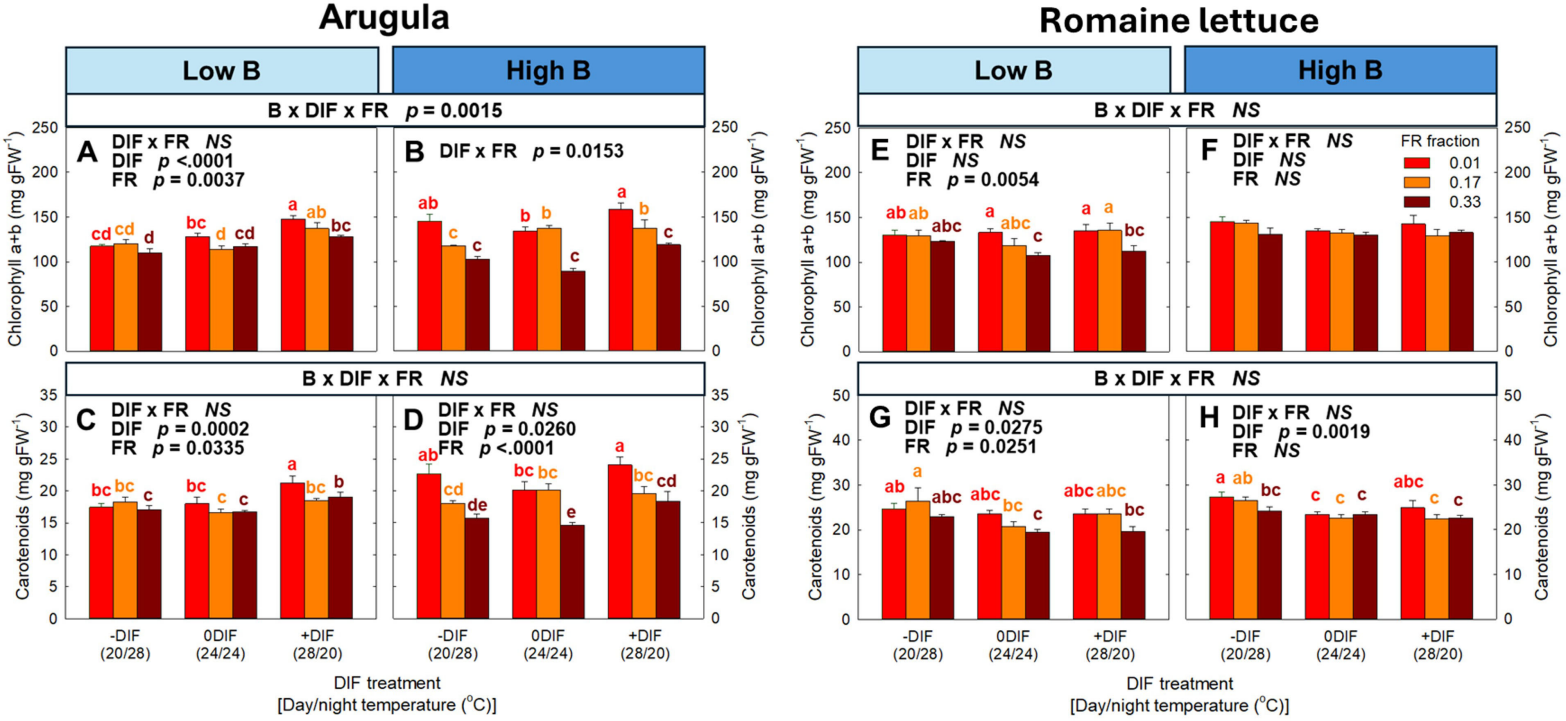
The interactive effect between light spectral quality [0.01, 0.17, and 0.33 of far-red (FR) fraction] and temperature [-DIF (difference between day and night temperature), 0DIF, +DIF] on chlorophyll a+b **(A, B)** and carotenoids **(C, D)** of arugula and chlorophyll a+b **(E, F)** and carotenoids **(G, H)** of romaine lettuce under low and high blue light conditions. Three-way ANOVA was conducted to assess the interactive effects among FR fraction, DIF, and blue light. To further evaluate the interactions between FR light and DIF under different blue light intensities, two-way ANOVA was performed for FR fraction and DIF within each blue light level. Different letters above the mean ± SE (n = 3 in the third replicate) indicate significant difference among the nine treatments (three FR fractions x three DIF treatments) at p < 0.05. NS stands for non-significance.

Photosynthetic pigments (i.e., chlorophyll a+b and carotenoids) showed no significant three-way interaction among FR light, DIF, and blue light in both arugula and romaine lettuce, except for chlorophyll a+b content in arugula (**Figure 5**). The photosynthetic pigments generally decrease with increasing FR fraction in both species. +DIF increased photosynthetic pigments in arugula while resulting in no changes or decreases in lettuce.

**Figure 6:**
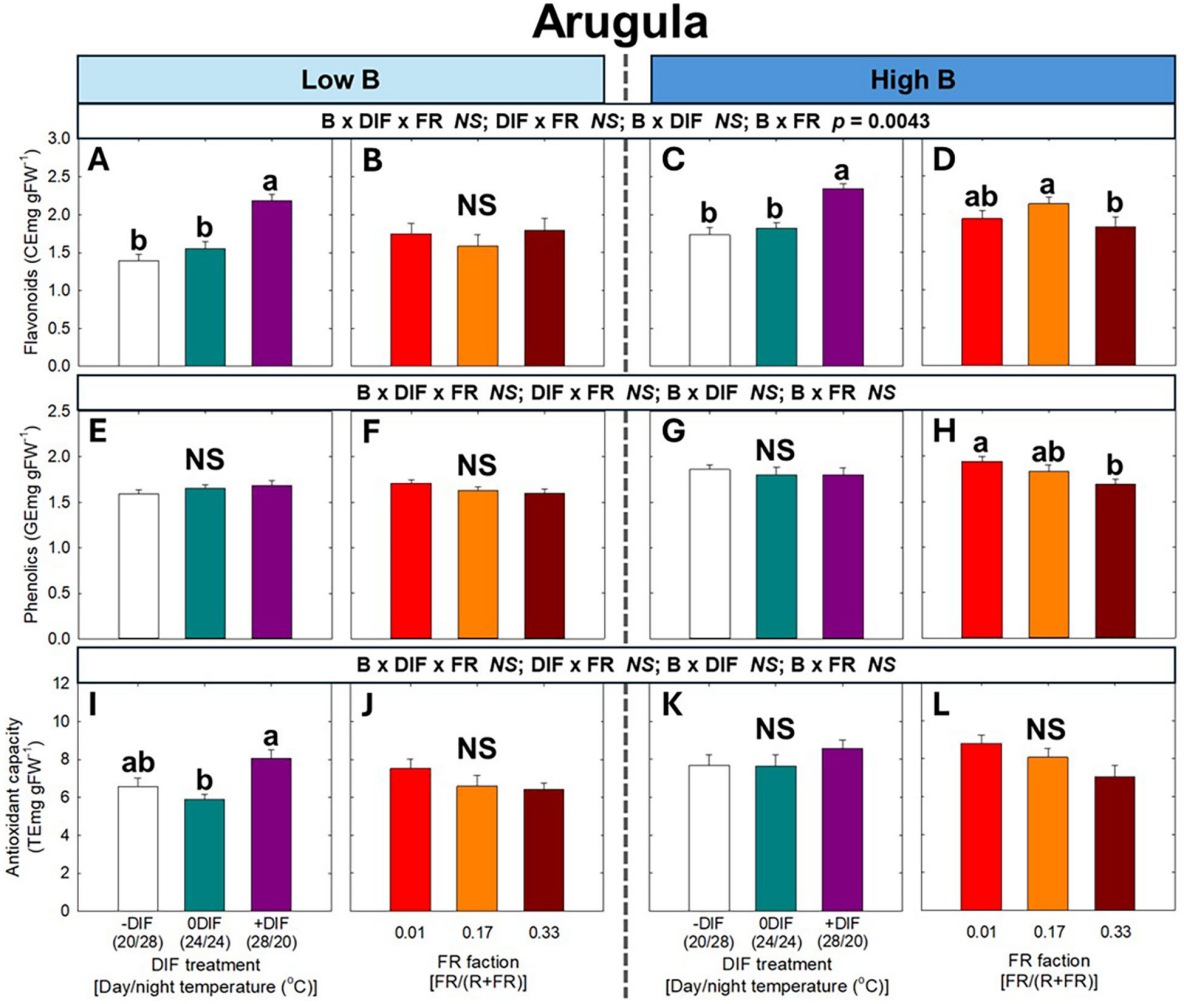
The interactive effect between light spectral quality [0.01, 0.17, and 0.33 of far-red (FR) fraction] and temperature [-DIF (difference between day and night temperature), 0DIF, +DIF] on flavonoids **(A-D)**, phenolics **(E-H)**, and antioxidant capacity **(I-L)** of arugula under low and high blue light conditions. Three-way ANOVA was conducted to assess the interactive effects among FR fraction, DIF, and blue light. To further evaluate the interactions between FR light and DIF under different blue light intensities, two-way ANOVA was performed for FR fraction and DIF within each blue light level. Different letters above the mean ± SE (n = 3 in the third replicate) indicate significant difference among the nine treatments (three FR fractions x three DIF treatments) at p < 0.05. NS stands for non-significance.

**Figure 7:**
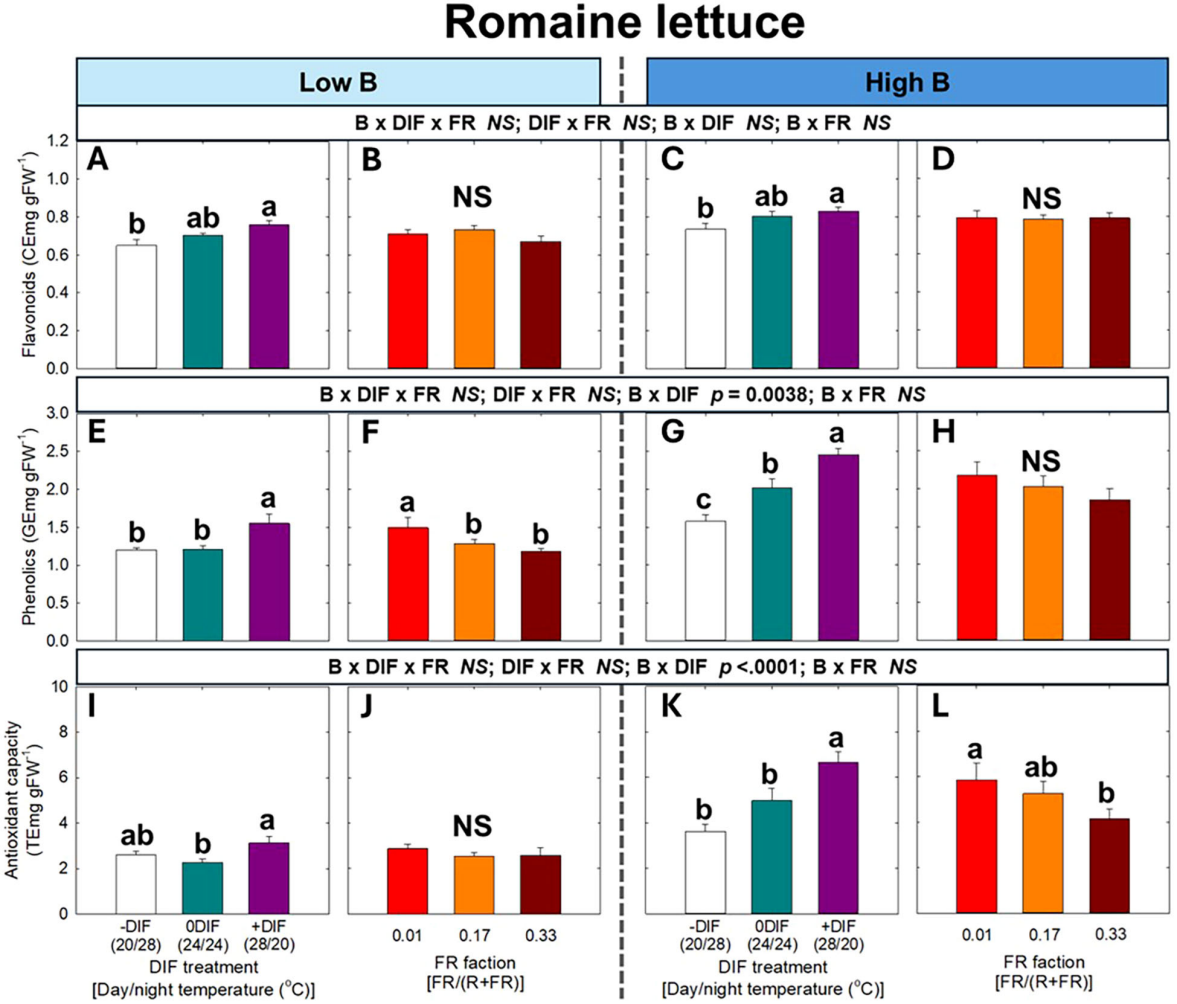
The interactive effect between light spectral quality [0.01, 0.17, and 0.33 of far-red (FR) fraction] and temperature [-DIF (difference between day and night temperature), 0DIF, +DIF] on flavonoids **(A-D)**, phenolics **(E-H)**, and antioxidant capacity **(I-L)** of romaine lettuce under low and high blue light conditions. Three-way ANOVA was conducted to assess the interactive effects among FR fraction, DIF, and blue light. Different letters above the mean ± SE (n = 3 in the third replicate) indicate significant difference among the nine treatments (three FR fractions x three DIF treatments) at p < 0.05. NS stands for non-significance.

For secondary metabolites and antioxidant capacity, no significant three-way interaction among FR light, DIF, and blue light was observed in either arugula or romaine lettuce (**Figures 6**, **7**). At both low and high blue light intensities, +DIF significantly increased flavonoid levels in arugula, and flavonoids, phenolics, and antioxidant capacity in romaine lettuce. By contrast, FR light significantly reduced phenolic content at high blue light in arugula, as well as phenolics at low blue and antioxidant capacity at high blue light in romaine lettuce.

## 4 Discussion

### 4.1 FR light and +DIF synergistically promoted stem elongation under low blue light but enhanced leaf expansion under high blue light

Our results indicated that FR light and DIF interact to regulate plant morphology, particularly leaf expansion and stem elongation in arugula and romaine lettuce (**Figures 2**, **3**; **Supplementary Figure 2**). However, these interactive effects were further influenced by blue light intensity. Notably, in arugula, the synergistic effect between FR light and +DIF occurred in different organs (i.e., leaves *versus* stems) depending on blue light levels. Specifically, FR light and +DIF synergistically increased total leaf area in arugula under high blue light, whereas under low blue light, the synergistic increase was observed in stem length (**Figures 3A–D**). Consistently, previous studies have shown that light intensity strongly modulated the responses to FR light in lettuce, with FR light promoting stem elongation at lower light intensity (100–200 µmol m^−2^ s^−1^) but enhancing leaf expansion at higher light intensity (300–500 µmol m^−2^ s^−1^) (Kusuma and Bugbee, 2023; Jeong et al., 2024c). These findings suggested that FR light and +DIF preferentially promote stem elongation under low blue light, whereas they enhanced leaf expansion under high blue light.

The shift of growth patterns in response to different environmental cues may be associated with distinct adaptative strategies to shade signals. Plants generally adopt two contrasting strategies: shade tolerance and shade avoidance (Valladares and Niinemets, 2008; Gommers et al., 2013). Shade-tolerant responses typically involve increased leaf expansion with reduced leaf thickness, whereas shade-avoiding responses often include enhanced hypocotyl, stem, and petiole elongation. Despite their contrasting morphological traits, both responses ultimately aim to maximize photon capture and thereby improve photosynthetic carbon gain (Valladares and Niinemets, 2008). Moreover, both strategies share a common signaling mechanism mediated by PHYs, and their differential response arise from the activities of negative regulators of PIFs, such as PHYA and LONG HYPOCOTYL IN FAR-RED LIGHT 1 (HFR1) (Molina-Contreras et al., 2019; Martinez-Garcia and Rodriguez-Concepcion, 2023). Given that phytochrome photoequilibrium (PPE) was comparable between blue light levels in this study (**Table 1**), the changes in PHY activity cannot explain the differential effects of FR light and +DIF on leaves and stems under different blue light intensities. A more plausible explanation is that blue light-activated CRYs suppressed PIF activity and associated hormonal signaling (Ma et al., 2016; Wang and Lin, 2020), maintaining shade-tolerant responses even under the combined effect of FR light and +DIF. These results indicate that blue light influences the adaptative strategy plants adopt in response to FR light and +DIF. This interpretation aligns with the findings of Shin and Runkle (2025); they found that increasing light intensity with R and FR light could not inhibit the shade responses induced by FR light on plant morphology, emphasizing that the suppression of FR light-mediated morphological responses at high light intensity may be primarily attributed to blue light rather than other spectral regions. These findings further suggest that manipulating the blue light intensity is a key strategy utilizing shade-tolerant leaf expansion in response to FR light under varying temperature conditions.

Similar to arugula, lettuce exhibited a diminished synergistic effect of FR light and +DIF on stem elongation as blue light increased (**Figures 3I, J**). However, unlike arugula, lettuce showed no synergistic increase in total leaf area in response to FR light and +DIF at either blue light level (**Figures 3G, H**). Consistently, biomass allocation patterns also diverged between the two species. Under high blue light, lettuce showed a significant decrease in leaf fraction when exposed to the combination of FR light and +DIF, but arugula maintained its leaf fraction (**Supplementary Figures 2A, B, I, J**). These results highlight species-specific sensitivity to FR light and temperature. The interspecific variation in shade sensitivity is well-documented, which is broadly categorized as shade-avoiding species and shade-tolerant species (Valladares and Niinemets, 2008; Demotes-Mainard et al., 2016). Beyond species-level differences, the magnitude of the spectral response can also vary among cultivars (Meng et al., 2019). Moreover, previous studies have reported a specific- and cultivar-specific response to the interactive effects of FR light and temperature (Jeong et al., 2024b; 2025). These results underscore the necessity of considering the traits of species and cultivars when regulating light spectrum and temperature to manipulate plant morphology.

### 4.2 Blue light intensity is critical to maintain the beneficial effect of FR light and +DIF on leaf expansion and crop yield

Because of the strong correlation with biomass accumulation, leaf expansion is a key determinant of crop yield through its contribution to photon capture and canopy photosynthesis (Kaiser et al., 2019; Zhen and Bugbee, 2020a). Accordingly, in horticultural production, substituting or supplementing FR light is frequently incorporated into LED lighting systems to stimulate leaf expansion and photon capture, thereby increasing biomass production across a wide range of vegetable and ornamental species (Park and Runkle, 2017; Meng and Runkle, 2019; Zhen and Bugbee, 2020a). Meanwhile, +DIF (warmer day than night) occurs either naturally through daytime heating or intentionally as part of temperature management to enhance photosynthetic efficiency and biomass accumulation (Kanno and Makino, 2010; Matsuda et al., 2014). Our study showed that FR light and +DIF synergistically stimulate morphological modification, expressed in different organs depending on blue light intensity (i.e., stem under low blue light *versus* leaf under high blue light) (**Figure 3**). These findings have two major implications in horticultural production. First, under low blue light, the coincidence of FR light and +DIF may unexpectedly reduce crop yield due to excessive stem elongation that limits leaf development and overall biomass accumulation (i.e., an enhanced shade-avoiding response). Second, under high blue light, the combination of FR light and +DIF effectively maximized crop yield through their synergistic enhancement of leaf expansion and consequent biomass accumulation (**Figures 3E, F, K, L**). For example, at +DIF, FR light increased total DW by 49% in arugula, whereas the enhancement was smaller (26%) at 0DIF and negligible at -DIF (**Figures 3E, F**). This synergistic promotion of leaf expansion occurred only when stem elongation was effectively suppressed by blue light, indicating that blue light intensity could play a critical role in determining whether the interactive effects between FR light and +DIF act as a growth-promoting or growth-limiting factor. Similarly, in lettuce, although a clear synergistic increase in leaf expansion was not observed, high blue light prevented the yield loss that could otherwise have occurred by the combination of FR light and +DIF under low blue light (**Figures 3K, L**). Taken together, these results emphasize that co-optimizing spectral composition and temperature regimes (i.e., DIF) is essential for maximizing biomass production and maintaining stable crop yield in controlled environmental agriculture.

### 4.3 Morphological and physiological acclimation to FR light and DIF altered leaf photosynthetic activity

FR light has been reported to synergistically enhance leaf photosynthetic efficiency when combined with shorter wavelengths, a phenomenon known as the Emerson enhancement effect (Emerson and Rabinowitch, 1960; Zhen and van Iersel, 2017). While FR light alone is largely ineffective in driving photosynthesis, its PSI-specific excitation becomes effective when paired with shorter wavelengths that primarily excite PSII (Hogewoning et al., 2012). By providing additional excitation to PSI, FR light helps to balance energy distribution between PSI and PSII, thereby ensuring a more efficient electron transport through the photosynthetic apparatus (Zhen and van Iersel, 2017; Zhen and Bugbee, 2020b). Consistently, our study showed that *Φ_PSII_* was significantly increased in both arugula and lettuce grown under higher FR light fractions compared with treatments lacking FR light (**Figures 4C, D, I, J**).

Despite the enhancement of photochemical efficiency, both species exhibited a decreasing trend in estimated daily carbon gain per unit leaf area under FR light (**Figures 4E, F, K, L**). This reduction was attributable to FR light-induced anatomical and physiological acclimation, particularly the increase in specific leaf area (i.e., thinner leaves) and the decrease in chlorophyll concentration (**Figure 5**; **Supplementary Figure 5**) (Chow et al., 1990; Li and Kubota, 2009). Thinner leaves restrict CO_2_ fixation capacity because reduced mesophyll thickness limits the number of chloroplasts per unit area, ultimately resulting in a lower maximum CO_2_ assimilation rate (Zou et al., 2019). Similarly, in our study, plants exposed to +DIF showed lower estimated carbon gain compared to both -DIF and 0DIF, which were likely associated with their thinner leaves (**Supplementary Figure 5**). In contrast, higher blue light increased the estimated daily carbon gain under the absence of FR light (**Figures 4E, F, K, L**). This may be also due to morphological and physiological acclimation, such as increased leaf thickness and enhanced photosynthetic pigments, to blue light (Hoffmann et al., 2016; Samuolienė et al., 2017; Kong and Nemali, 2021).

However, it should be noted that the estimated daily carbon gain under each treatment was derived from the photosynthetic measurement at single-leaf level. Single-leaf photosynthetic measurement often does not align with actual crop yield (Evans, 1975; Elmore, 1980). Discrepancy is particularly evident under conditions where plant morphology is substantially altered, indicating that biomass accumulation (i.e., final carbon gain) primarily depends on photon capture rather than single-leaf photosynthesis (Jeong et al., 2024c). Consistently, although plants acclimated to FR light typically showed a reduced single-leaf photosynthetic rate, FR light could maintain equivalent carbon gain at the canopy level by enhancing photon capture (Zhen and Bugbee, 2020a). These findings explain the mismatch between the estimated carbon gain per unit leaf area and the final biomass observed in our study (**Figures 3**, **4**).

### 4.4 +DIF enhanced secondary metabolite accumulation and antioxidant capacity, while FR light tended to reduce these parameters, with both effects independent of blue light intensity

One of the main production objectives in indoor farming systems is to enhance health-promoting compounds, such as secondary metabolites (Appolloni et al., 2022; Bafort and Jijakli, 2024). Blue light is an effective stimulus for promoting the accumulation of various secondary metabolites and enhancing antioxidant capacity (Zhang et al., 2021). In our study, blue light generally increased secondary metabolite contents and antioxidant capacity in the absence of FR light (**Figures 6**, **7**). However, unlike its effects on morphological and growth traits, blue light did not further modify the effects of FR light and DIF on secondary metabolite accumulation and antioxidant capacity. For example, FR light reduced photosynthetic pigments and/or secondary metabolites, resulting in lower nutritional quality of leafy greens at any blue light level (**Figures 6**, **7**). The decrease in pigment and secondary metabolite accumulation under FR light is often attributed to the dilution effect, which occurs alongside enhanced leaf expansion (Kong and Nemali, 2021; Li and Kubota, 2009). The reduction in these phytochemicals may also result from FR light downregulating the expression of key biosynthetic genes through the PHY-mediated signaling network (Toledo-Ortiz et al., 2010).

The FR light-mediated decrease in secondary metabolite levels could be compensated by utilizing +DIF treatments (**Figures 6**, **7**). Given that secondary metabolites are produced in response to environmental stressors, the daily temperature fluctuation might function as a stress signal that modifies metabolic pathways in plants (Al Jaouni et al., 2018; Pandey et al., 2018; Pant et al., 2021). However, in our study, pigments and secondary metabolite contents and antioxidant capacity under -DIF were similar to, or even lower than, those under 0DIF (**Figures 6**, **7**). Moreover, the F_v_/F_m_, a widely used indicator of plant stress, consistently ranged from the typical range of healthy plants (0.79 to 0.84 in both arugula and lettuce), which means plants have fully acclimated to the experimental environments (**Figures 4A, B, G, H**) (Maxwell and Johnson, 2000). This result suggests that the environmental stress imposed by daily temperature fluctuation was not a major factor for the observed changes in the accumulation of pigments and secondary metabolites and antioxidant capacity. Instead, the enhanced secondary metabolite contents under +DIF may be associated with the more efficient use of photosynthates, as DIF can influence photosynthetic carbon fixation and utilization by regulating the balance between daytime photosynthesis and nighttime respiration. Specifically, compared with –DIF (cooler day/warmer night), +DIF (warmer day/cooler night) generally enhances carbon gain because higher daytime temperatures stimulate rubisco activity and other photosynthetic enzymes, while cooler nights suppress respiration (Matsuda et al., 2012, 2014). This improved carbon balance under +DIF not only supports biomass accumulation but may also increase the pool of carbon skeletons available for secondary metabolite synthesis, thereby contributing to enhanced production of antioxidants.

## 5 Conclusion

FR light, DIF, and blue light interactively influenced plant morphology, biomass accumulation, and secondary metabolite production in arugula and romaine lettuce. The interactive effects of FR light and DIF were strongly dependent on blue light intensity. These results demonstrate that blue light is a key determinant of whether the interactions between FR light and DIF are beneficial or detrimental to crop growth. From a practical perspective, these findings provide valuable insights for optimizing environmental control strategies in controlled-environment agriculture. Under low blue light, the coexistence of FR light and +DIF may lead to unexpected yield reductions due to excessive stem elongation and restricted leaf development. In contrast, under high blue light conditions, the combination of FR light and +DIF synergistically enhanced leaf expansion, resulting in greater biomass accumulation. Moreover, these interactive responses were species-specific. For example, in arugula, high blue light suppressed excessive stem elongation and enabled synergistic increases in leaf expansion and biomass, whereas under low blue light, FR light and +DIF caused excessive stem elongation with limited leaf expansion. In lettuce, synergistic promotion of leaf expansion was not observed at either blue light level; instead, high blue light effectively inhibited stem elongation and thereby preserved the positive effects of FR light and +DIF on leaf growth and final biomass. Regarding nutritional quality, the reduced secondary metabolite level by FR light could be compensated for by +DIF and higher blue light intensity. Overall, our findings suggest that the combination of FR light, +DIF, and high blue light intensity can be an effective strategy to maximize both crop yield and quality in indoor farming.

## Data Availability

The raw data supporting the conclusions of this article will be made available by the authors, without undue reservation.
