# Supplementary material for: Blue light modulates the interactive effects of far-red light and day–night temperature difference on the growth, morphology and physiology of arugula and lettuce
Source: Front Plant Sci. 2025 Dec 3;16:1721040. doi: 10.3389/fpls.2025.1721040 (PMC12709603; doi:10.3389/fpls.2025.1721040)
Supplement: Supplementary file 1 [file DataSheet1.docx]

Supplementary Material

# Supplementary Figures


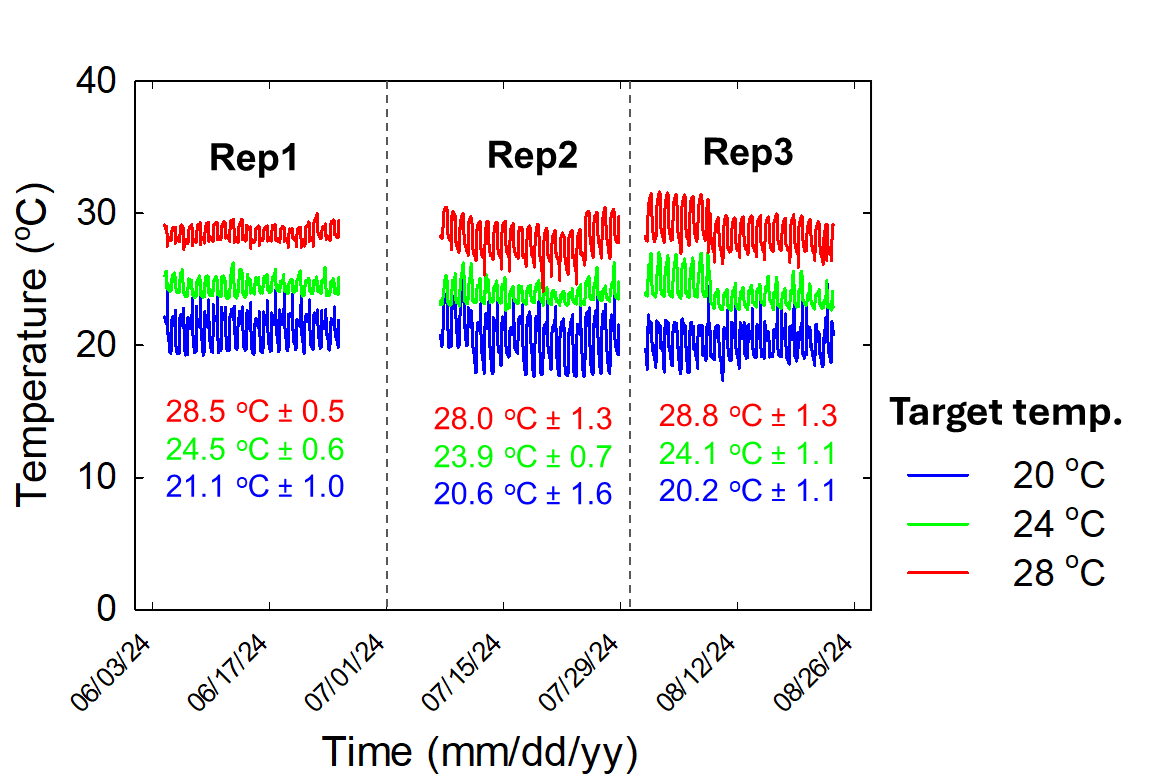


**Supplementary Figure 1.** The measured temperature (± standard deviation) of growth chambers set to 20, 24, and 28 ℃ across three experimental replicates. Among the three day-night temperature difference (DIF) treatments, two treatments (i.e., -8 DIF and +8 DIF) were created by transferring plants between the 20 ℃ and 28 ℃ growth chambers twice daily: once within 30 minutes before the start of the light period and again at the beginning of the dark period. Plants under 0 DIF treatment were maintained in the 24 ℃ chamber.


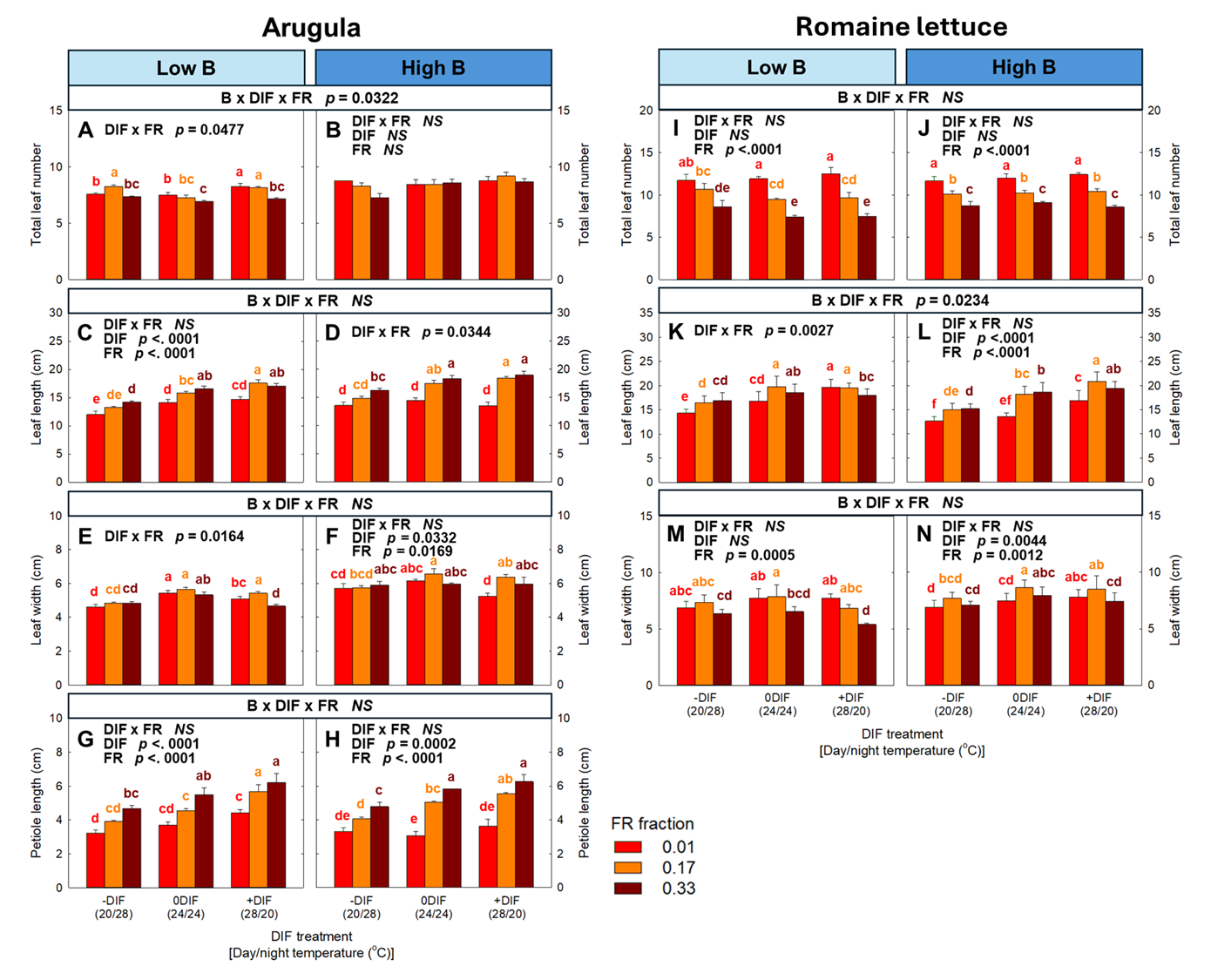


**Supplementary Figure 2.** The interactive effect between light spectral quality [0.01, 0.17, and 0.33 of far-red (FR) fraction] and temperature [-DIF (difference between day and night temperature), 0DIF, +DIF] on total leaf number (A-B), leaf length (C-D), leaf width (E-F), and petiole length (G-H) of arugula and total leaf area (I-J), leaf length (K-L), and leaf width (M-N) of romaine lettuce under low and high blue light conditions. Three-way ANOVA was conducted to assess the interactive effects among FR fraction, DIF, and blue light. To further evaluate the interactions between FR light and DIF under different blue light intensities, two-way ANOVA was performed for FR fraction and DIF within each blue light level. Different letters above the mean ± SE [n = 3; subsamples (4 plants per treatment per replicate study) were averaged before statistical analysis] indicate significant difference among the nine treatments (three FR fractions x three DIF treatments) at p < 0.05. NS stands for non-significance.


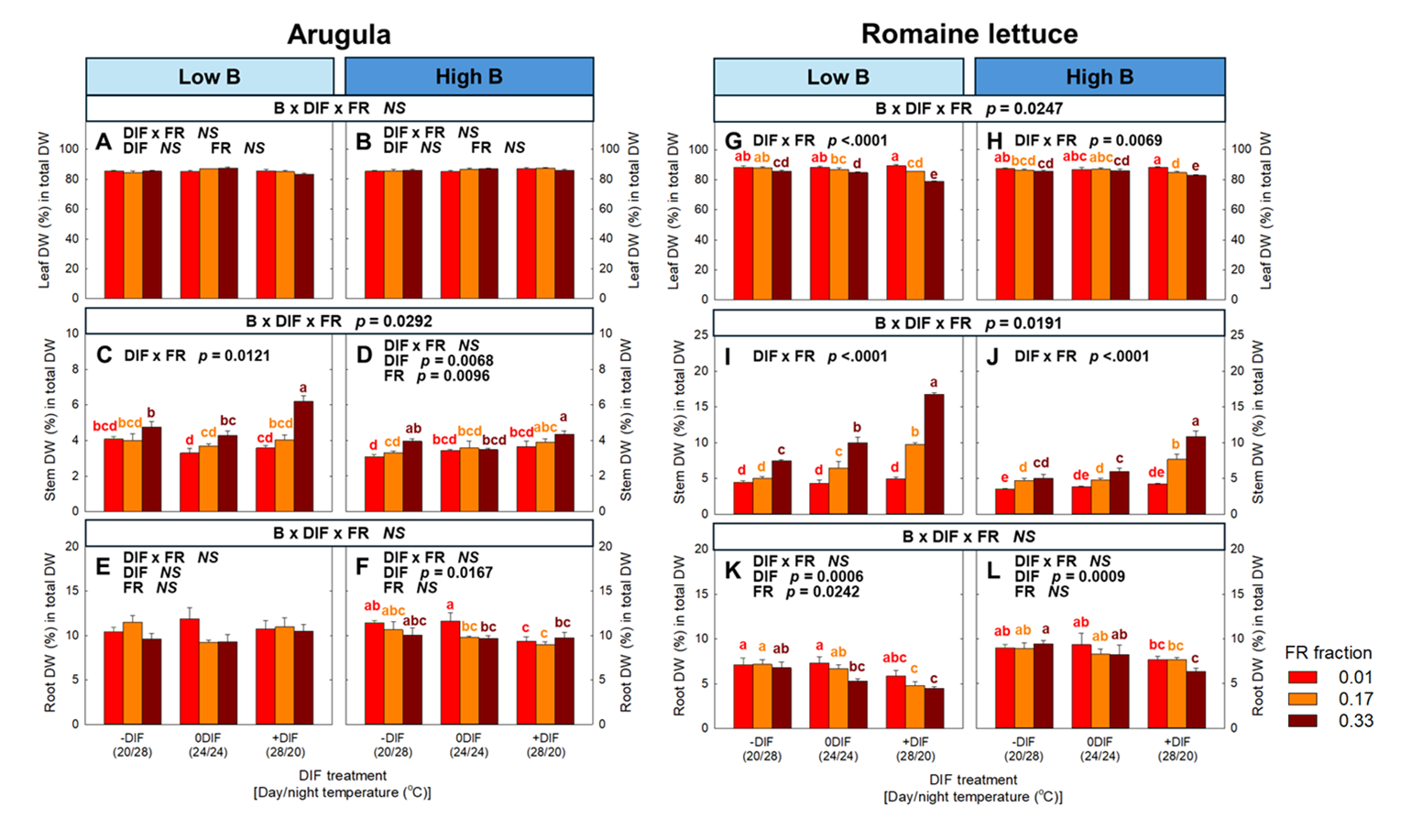


**Supplementary Figure 3.** The interactive effect between light spectral quality [0.01, 0.17, and 0.33 of far-red (FR) fraction] and temperature [-DIF (difference between day and night temperature), 0DIF, +DIF] on leaf dry weight (DW) (%) in total DW (A-B), stem DW (%) in total DW (C-D), and root DW (%) in total DW (E-F) of arugula and leaf DW in total DW (%) in total DW (G-H), stem DW (%) in total DW (I-J), and root DW (%) in total DW (K-L) of romaine lettuce under low and high blue light conditions. Three-way ANOVA was conducted to assess the interactive effects among FR fraction, DIF, and blue light. To further evaluate the interactions between FR light and DIF under different blue light intensities, two-way ANOVA was performed for FR fraction and DIF within each blue light level. Different letters above the mean ± SE [n = 3; subsamples (4 plants per treatment per replicate study) were averaged before statistical analysis] indicate significant difference among the nine treatments (three FR fractions x three DIF treatments) at p < 0.05. NS stands for non-significance.


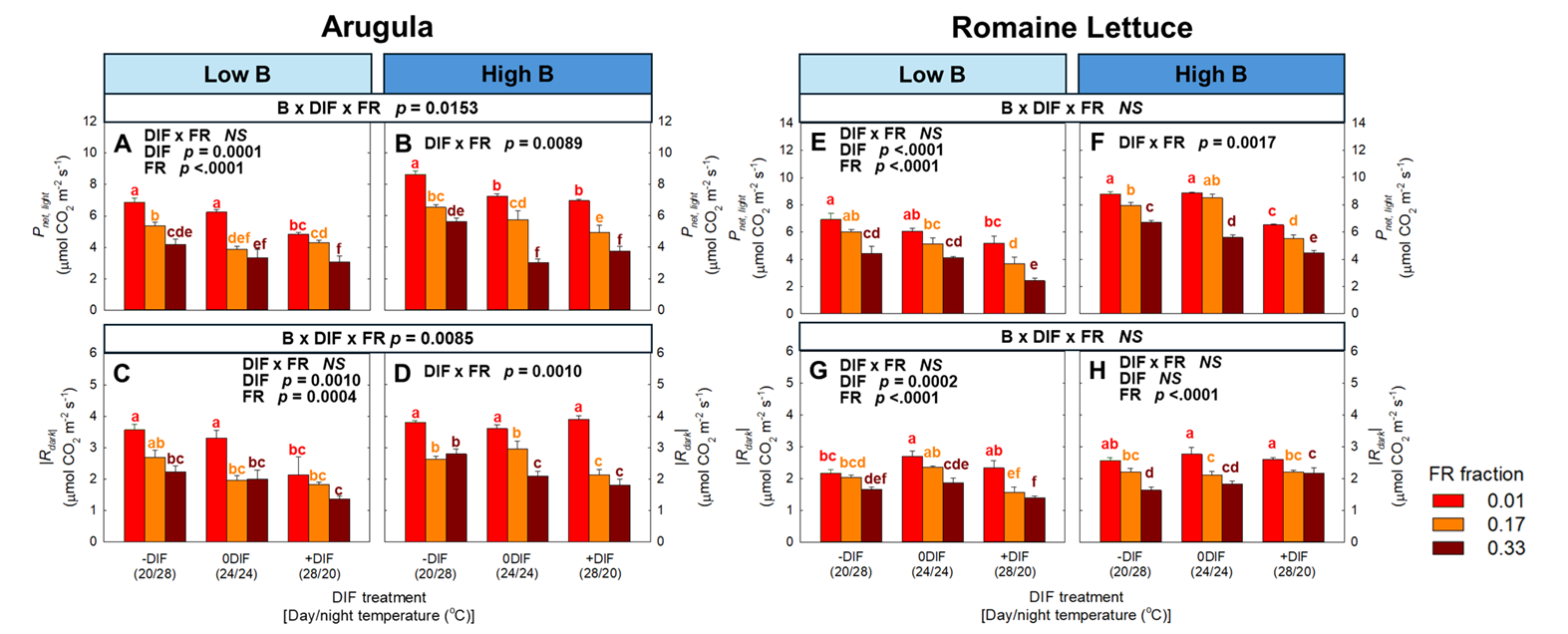


**Supplementary Figure 4.** The interactive effect between light spectral quality [0.01, 0.17, and 0.33 of far-red (FR) fraction] and temperature [-DIF (difference between day and night temperature), 0DIF, +DIF] on *P_net, light_* (A-B) and *R_dark_* (C-D) of arugula and *P_net, light_* (E-F) and *R_dark_* (G-H) of romaine lettuce under low and high blue light conditions. Three-way ANOVA was conducted to assess the interactive effects among FR fraction, DIF, and blue light. To further evaluate the interactions between FR light and DIF under different blue light intensities, two-way ANOVA was performed for FR fraction and DIF within each blue light level. Different letters above the mean ± (n = 3 in the third replicate) indicate significant difference among the nine treatments (three FR fractions x three DIF treatments) at p < 0.05. NS stands for non-significance.


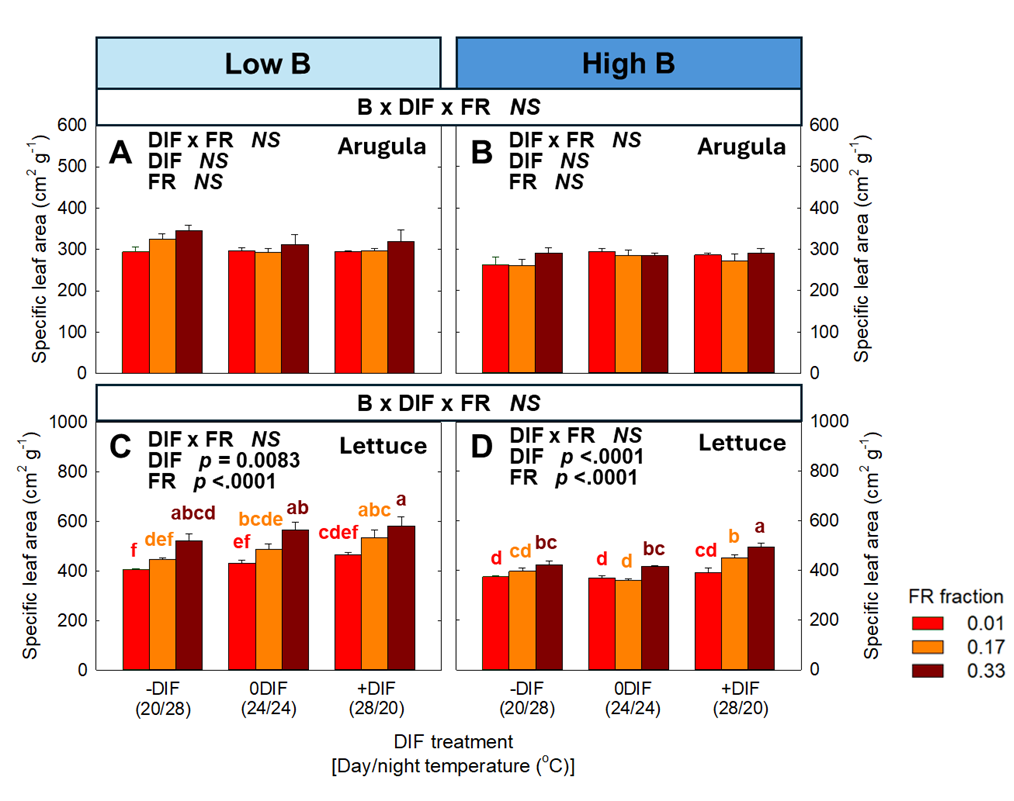


**Supplementary Figure 5.** The interactive effect between light spectral quality [0.01, 0.17, and 0.33 of far-red (FR) fraction] and temperature [-DIF (difference between day and night temperature), 0DIF, +DIF] on specific leaf area of arugula (A-B) and romaine lettuce (C-D) under low and under high blue light conditions. Three-way ANOVA was conducted to assess the interactive effects among FR fraction, DIF, and blue light. To further evaluate the interactions between FR light and DIF under different blue light intensities, two-way ANOVA was performed for FR fraction and DIF within each blue light level. Different letters above the mean ± SE [n = 3; subsamples (4 plants per treatment per replicate study) were averaged before statistical analysis] indicate significant difference among the nine treatments (three FR fractions x three DIF treatments) at p < 0.05. NS stands for non-significance.
